# Loss-of-function/gain-of-function polymorphisms of the ATP sensitive P2X7R influence sepsis, septic shock, pneumonia, and survival outcomes

**DOI:** 10.3389/fimmu.2024.1352789

**Published:** 2024-06-20

**Authors:** Johanna Guggemos, Stephen J. Fuller, Kristen K. Skarratt, Benjamin Mayer, E. Marion Schneider

**Affiliations:** ^1^ Clinic for Anesthesiology and Intensive Care Medicine, Ulm University Hospital, Ulm, Germany; ^2^ Nepean Clinical School, Faculty of Medicine and Health, The University of Sydney, Kingswood, NSW, Australia; ^3^ Department of Haematology, Nepean Hospital, Penrith, NSW, Australia; ^4^ Institute for Epidemiology and Medical Biometry, Ulm University, Ulm, Germany

**Keywords:** P2RX7, loss-of-function (LOF), gain-of-function (GOF) single nucleotide polymorphisms (SNP), linkage disequilibrium, pneumonia, sepsis, septic shock, ATP

## Abstract

**Introduction:**

Extracellular ATP (eATP) released from damaged cells activates the P2X7 receptor (P2X7R) ion channel on the surface of surrounding cells, resulting in calcium influx, potassium efflux and inflammasome activation. Inherited changes in the P2X7R gene (*P2RX7*) influence eATP induced responses. Single nucleotide polymorphisms (SNPs) of *P2RX7* influence both function and signaling of the receptor, that in addition to ion flux includes pathogen control and immunity.

**Methods:**

Subjects (n = 105) were admitted to the ICU at the University Hospital Ulm, Germany between June 2018 and August 2019. Of these, subjects with a diagnosis of sepsis (n = 75), were also diagnosed with septic shock (n = 24), and/or pneumonia (n = 42). Subjects with pneumonia (n = 43) included those without sepsis (n = 1), sepsis without shock (n = 29) and pneumonia with septic shock (n = 13). Out of the 75 sepsis/septic shock patients, 33 patients were not diagnosed with pneumonia. Controls (n = 30) were recruited to the study from trauma patients and surgical patients without sepsis, septic shock, or pneumonia. SNP frequencies were determined for 16 *P2RX7* SNPs known to affect P2X7R function, and association studies were performed between frequencies of these SNPs in sepsis, septic shock, and pneumonia compared to controls.

**Results:**

The loss-of-function (LOF) SNP rs17525809 (T253C) was found more frequently in patients with septic shock, and non-septic trauma patients when compared to sepsis. The LOF SNP rs2230911 (C1096G) was found to be more frequent in patients with sepsis and septic shock than in non-septic trauma patients. The frequencies of these SNPs were even higher in sepsis and septic patients with pneumonia. The current study also confirmed a previous study by our group that showed a five SNP combination that included the GOF SNPs rs208294 (C489T) and rs2230912 (Q460R) that was designated #21211 was associated with increased odds of survival in severe sepsis.

**Discussion:**

The results found an association between expression of LOF *P2RX7* SNPs and presentation to the ICU with sepsis, and septic shock compared to control ICU patients. Furthermore, frequencies of LOF SNPs were found to be higher in sepsis patients with pneumonia compared to those without pneumonia. In addition, a five SNP GOF combination was associated with increased odds of survival in severe sepsis. These results suggest that *P2RX7* is required to control infection in pneumonia and that inheritance of LOF variants increases the risk of sepsis when associated with pneumonia. This study confirms that *P2RX7* genotyping in pneumonia may identify patients at risk of developing sepsis. The study also identifies P2X7R as a target in sepsis associated with an excessive immune response in subjects with GOF SNP combinations.

## 1 Introduction

The clinical course of critically ill patients requiring intensive care involves systemic inflammation to clear pathogens followed by profound immunosuppression to allow healing and recovery. Tissue damage and bacterial or viral infections trigger upregulation and activation of the pattern-recognition receptor (PRR)/danger-associated molecular pattern (DAMP) receptor, P2X7 receptor (P2X7R) which activates the inflammasome and subsequent cytokine release. This characterizes sepsis as a disease with multifactorial debilitating conditions and loss of immune control to nosocomial and community-acquired infections with a high risk of death. The imbalance of the immune response appears to be responsible for the high risk of mortality in patients with sepsis, making it a major problem for intensive care units worldwide.

In 1972, Burnstock introduced the concept of purinergic signaling, describing adenosine triphosphate (ATP) as a major stressor for cells and tissues. In contrast, its metabolite adenosine, generated by cell surface expressed nucleotidases, was found to be a major immunosuppressor (1). ATP release occurs due to tissue damage, hypoxia, mechanical stimulation, changes in osmotic pressure (2), surgery (3), and hemorrhage (4). The most severe form of sepsis, septic shock, is associated with failure of the vascular system and metabolic insufficiency, resulting in hypotension and elevated serum lactate (5). In addition to its role in inflammation and immune dysfunction, P2X7R has been shown to be an essential receptor for macrophage-mediated bacterial killing (6). Furthermore, recent evidence suggests that P2X7R stimulation may modulate the host response by dampening inflammation by stimulating CD14 release from macrophages (7).

The controversial molecular evidence for P2X7R influencing tissue damage, inflammation, and immune dysfunction may be related in part to its high numbers of genetic polymorphisms including both loss-of-function (LOF) and gain-of-function (GOF) single nucleotide polymorphisms (SNPs) (8). Several studies have investigated the effect of SNP characteristics and their effect on P2X7R ion channel function and pro-inflammatory cytokine release (9–11). In addition to SNPs, the receptor can be modified by alternative splicing events (12–14).

P2X7R consists of three P2X7 protein subunits and is found on the membranes of cell surfaces and intracellular organelles (15). Inflammatory conditions can differentially modulate the membrane expression of P2X7 and affect eATP signaling in defined phases of sepsis (16).

In one of our previous studies, combinations of LOF and GOF SNPs were identified in patients with sepsis and in patients with virus-induced hemophagocytic syndromes which appear to be related to better survival and higher frequencies in patients with hemophagocytic lymphohistiocytosis (HLH) (17).

The current genetic association study measured the allele frequencies of LOF and GOF SNPs of the P2X7 gene (*P2RX7*) in intensive care unit (ICU) patients who were either non-septic or suffered from sepsis, septic shock, with and without pneumonia. It was hypothesized that LOF *P2RX7* SNPs might protect against excessive systemic inflammation and sepsis, but at the expense of pathogen clearance, whereas GOF *P2RX7* SNPs might be linked to better pathogen control, but increased risk of sepsis.

## 2 Materials and methods

### 2.1 Study population

**Figure 1:**
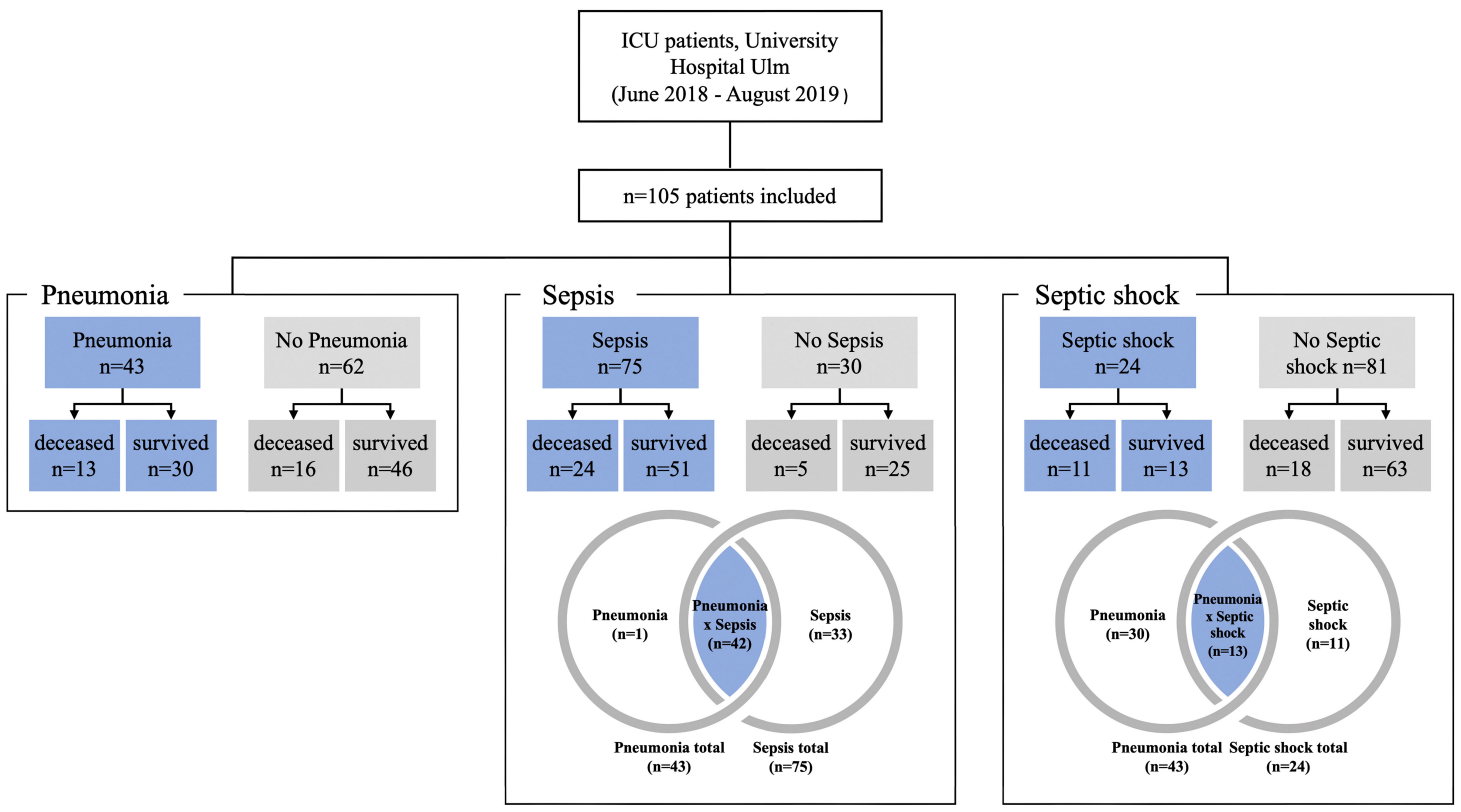
Subject and control groupings for analysis. Patients with pneumonia, sepsis or septic shock are shown as blue fields and the corresponding controls are shown in grey.

This study was approved by the Ethics Committee of Ulm University (application no. 284/18). Subjects (n = 105) for the study were recruited from the anesthesiologic ICU at the University Hospital Ulm, Germany between June 2018 and August 2019 (see **Figure 1**). Subjects were aged between 18 and 84 years and there were 77 males and 28 females. The mean age of the patients was 61.06 years (SD 15.82 years). Clinical information for each patient included is given in **Supplementary Table 1**, including information on co-morbidities, sterile trauma/hemorrhage, obesity, diabetes type 2, malignancies, chronic obstructive pulmonary disease (COPD), asthma, alcohol or nicotine abuse, organ dysfunction syndromes, as well as infections before manifesting critical illness. Thirty patients were non-septic, 75 patients were diagnosed with sepsis, of which 24 patients were in septic shock (**Figure 1**). Forty-two patients had pneumonia and sepsis including 13 patients with septic shock. Out of the 75 sepsis/septic shock patients, 33 patients did not suffer from pneumonia (**Figure 1**).

Non-septic patients comprised two major groups: i) patients with polytrauma (n=9) and ii) patients with major surgical interventions (n=25) including tumor resection (n=2), surgery related to a vascular disease (n=9) or neurosurgery (n=6). A total of n=6 patients from the polytrauma group underwent surgery during their stay in the ICU. The remaining three patients underwent surgery for hemorrhagic shock, compartment syndrome, and atypical gastric resection due to obesity. There were only two patients who did not belong to one of the two main groups: One patient was monitored in the ICU for Stanford type B aortic dissection, and the other patient had a bowel obstruction that did not require surgery (see **Supplementary Table 1** for more details).

### 2.2 Study design, DNA isolation, and genotyping

Following the patients’ characteristics given in **Figure 1**, a total of 105 patients were studied. The first step was to determine how many patients had sepsis (n=75) and how many did not have sepsis (n=30). Using these two groups, we investigated whether there were statistical differences with regard to the genetics of *P2RX7*. Subsequently, we studied for differences in *P2RX7* SNP frequencies in patients with septic shock (n=24) and patients without septic shock (n=81). Finally, this study calculated *P2RX7* SNP frequencies for all patients with pneumonia (n=43) versus patients without pneumonia (n=62) **Figure 1**.

**Figure 2:**
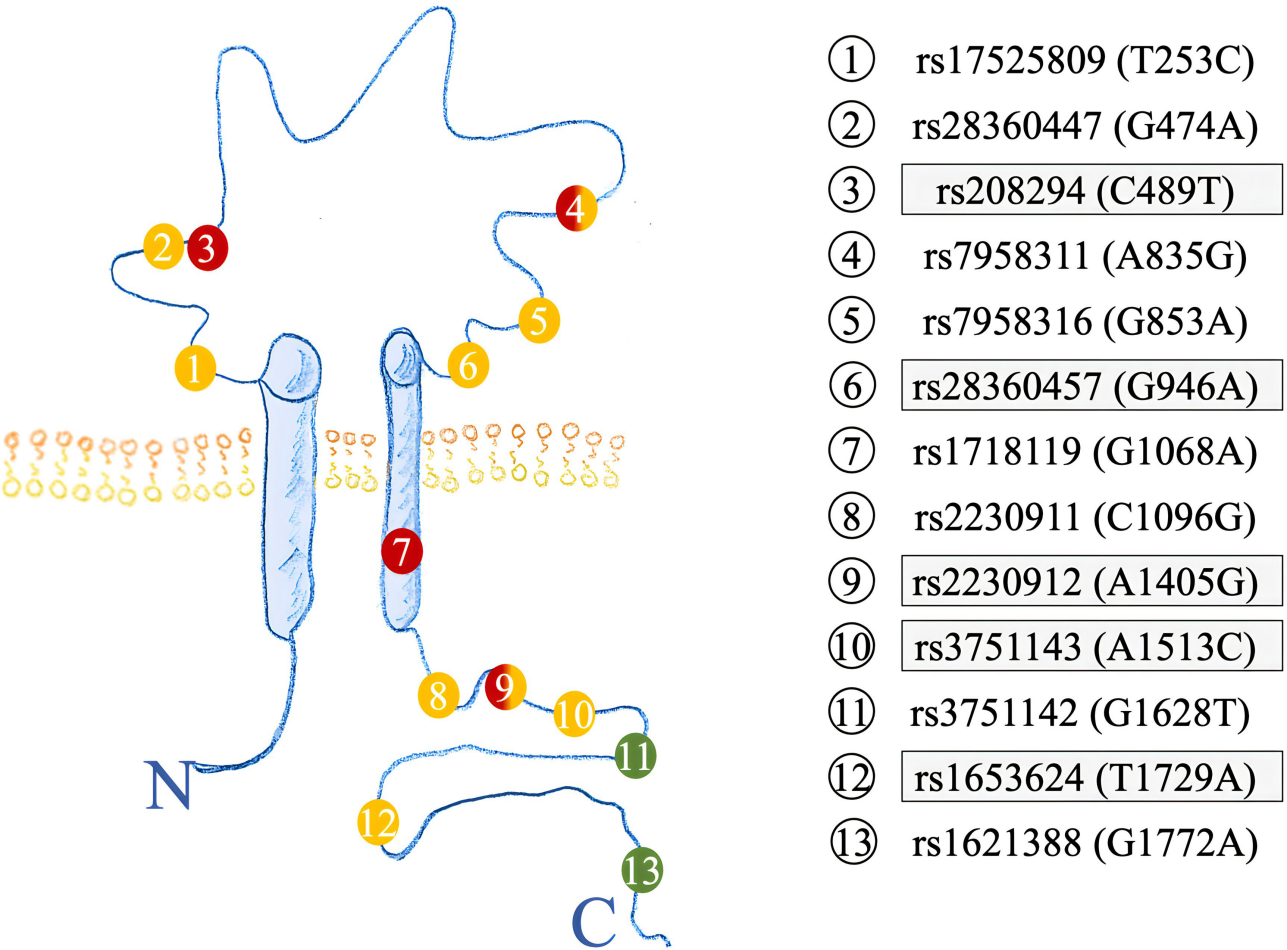
Structure of the P2X7R highlighting the amino acid location of SNPs investigated. Previous SNPs analyzed in (17) are highlighted in grey. GOF SNPs are red, LOF SNPs yellow, and synonymous SNPs colored in green.

**Figure 3:**
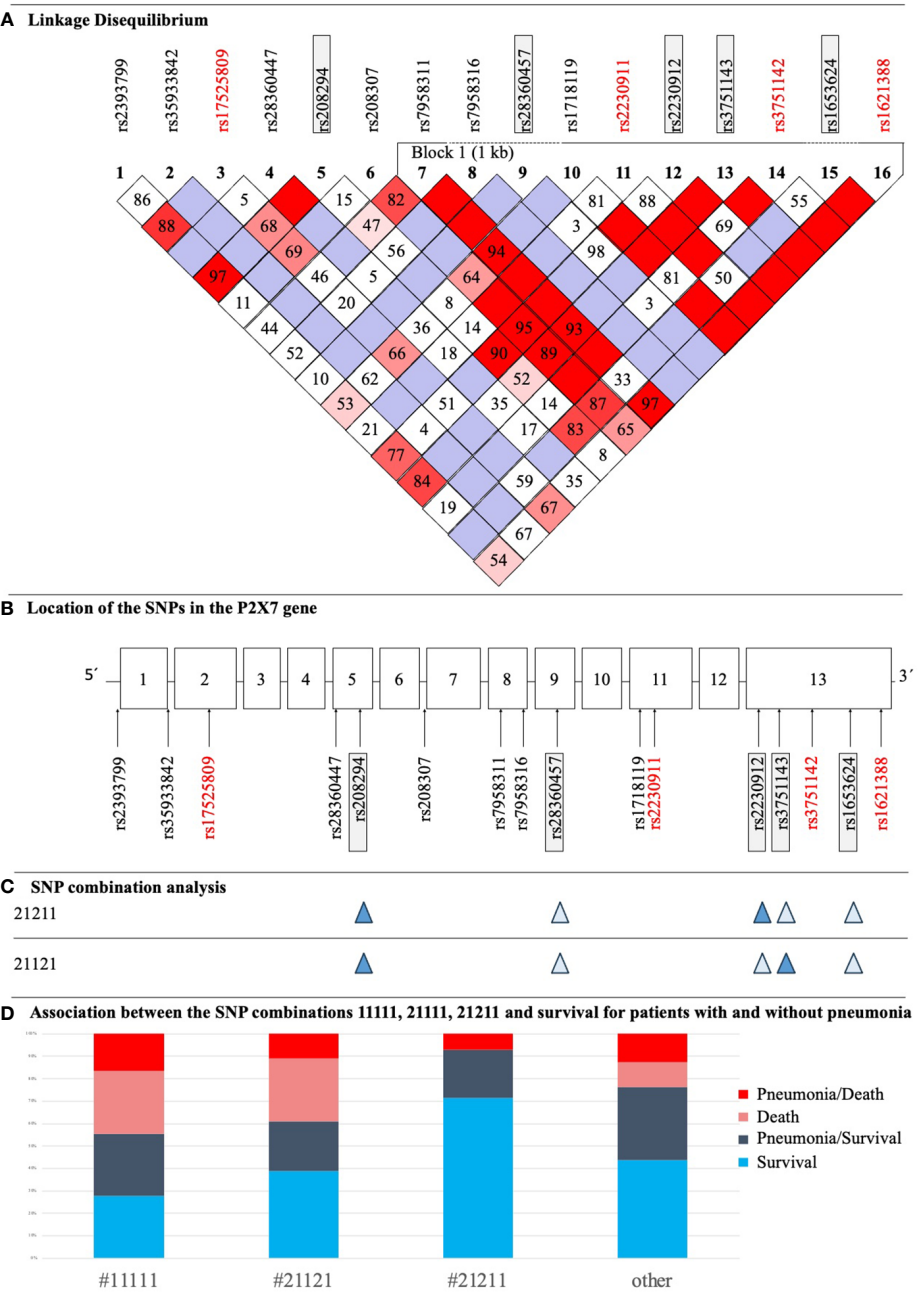
**(A)** LD Plot of *P2RX7*. The display was performed according to the Broad Institute’s Standard Color Scheme. The number in the boxes refers to the coefficient D’. SNPs that showed associations with pneumonia and/or sepsis are in red. **(B)** Structure of the *P2RX7* gene with the location of studied SNPs. SNPs with statistically significant results in the current study are highlighted red, SNPs being part of the SNP combination model are highlighted in grey boxes. **(C)** Illustration of two important SNP combinations related to survival, filled symbols indicate heterozygosity and open symbols indicate wildtype genotypes of the respective SNP. **(D)** Distribution of patients with sepsis with and without pneumonia, who survived/died and their corresponding P2RX7 SNP combinations as defined in a previous study (17).

Semi-automated DNA purification was done by using a Maxwell 16® LEV Blood DNA Kit and a Maxwell 16® (#AS1290, Promega®) (https://www.promega.com) instrument. A total of 300 µl of fresh EDTA blood from each patient (pseudonymized by a KeyPat-Id number), was mixed with 300 µl of Promega lysis buffer plus 30 µl Proteinase K (supplied in the kit). The samples were then vortexed for 10 seconds and incubated at 56°C for 20 min using a water bath. The high temperature and the detergents guanidinium thiocyanate (50-75%) and polyethylene glycol tert-octyl-phenyl ether (Triton-X, < 2%), which are components of the lysis buffer, lead to lysis of cell and nuclear membranes. The proteinase K degrades various proteins. Histones are also degraded, enabling the release of genomic DNA (gDNA). After the incubation time, the lysed blood sample was pipetted into well No.1 of the Maxwell 16® cartridge. The cartridges were placed into the cartridge holder with a plunger and an additional tube, containing 60 µl elution buffer. The cartridge holder was then placed inside the Maxwell 16® Instrument and the machine was started. The Maxwell 16® instrument uses paramagnetic particles, the MagnaCel™ particle, which takes advantage of the cellulose binding capacity of nucleic acids. During the automated purification process, gDNA is bound to the paramagnetic particle, washed with ethanol and eventually released into the elution buffer. Absorbance measurements determined the grade of genomic deoxyribonucleic acid (gDNA) using the NanoDrop® 1000 version 3.8.1 (https://www.thermofisher.com). After DNA isolation, 16 *P2RX7* SNPs (**Table 1**, **Figures 2**, **3A**) were genotyped using MassARRAY® technology following the manufacturers advice from Agena Bioscience (https://www.agenabio.com).

**Table 1:** Visualization of the investigated SNPs with location in the gene/protein, the change in the amino acid chain and the impact on receptor function.

dbSNP ID	amino acid change	position	function
**rs2393799 (C-762T)**	–	promotor	Decreased receptor expression (18)
**rs35933842 (G151 + 1T)**	-	intron 1	Splice site mutation, P2X7 null allele (14)
**rs17525809 (T253C)**	Val-76>Ala	exon 2	Partial LOF (11, 19)
**rs28360447 (G474A)**	Gly-150>Arg	exon 5	LOF (11)
**rs208294 (C489T)**	His-155>Tyr	exon 5	GOF (20)
**rs208307 (C641-5G)**	-	intron 6	Promotes exon 7, 8 skipping, null function (13)
**rs7958311 (G835A)**	His-270>Arg	exon 8	Remains unclear [LOF (21), GOF (11)]
**rs7958316 (G853A)**	Arg-276>His	exon 8	LOF (11)
**rs28360457 (G946A)**	Arg-307>Gln	exon 9	LOF (22)
**rs1718119 (G1068A)**	Ala-348>Thr	exon 11	GOF (11)
**rs2230911 (C1096G)**	Thr-357>Ser	exon 11	LOF (21)
**rs2230912 (A1405G)**	Gln-460>Arg	exon 13	Remains unclear [LOF (11), GOF (23)]
**rs3751143 (A1513C)**	Glu-496>Ala	exon 13	LOF (9, 19)
**rs3751142 (G1628T)**	Leu534Leu	exon 13	Synonymous variant (24)
**rs1653624 (T1729A)**	Ile-568>Asn	exon 13	LOF (19, 25)
**rs1621388 (G1772A)**	Pro582Pro	exon 13	Synonymous variant (24, 26)

### 2.3 Statistical analysis

IBM SPSS® version 26 was used for statistical data analysis with an α<0.05 level of significance used for all calculations. Patient characteristics such as age, sex, or length of stay were first tested for normal distribution using the Kolmogorov-Smirnov and Shapiro-Wilk tests. Because the data were not normally distributed, differences between mean tendencies were calculated using the Mann-Whitney U test. Odds ratios (OR) and 95% confidence intervals (CI) were reported for significant results.

All *P2RX7* SNPs (n=16) were first tested for deviations from Hardy Weinberg Equilibrium (HWE). Next, linkage disequilibrium (LD) was determined for all SNPs using Haploview version 4.2 and analyzed according to the Broad Institute’s standard coloring scheme (27). A logarithm of the odds (LOD) score ≥2 between two SNPs indicates genetic linkage, as opposed to a random result. In accordance with literature recommendations, SNPs with minor allele frequency (MAF) <1% were excluded from further calculations (28). To test for sample-to-population consistency, MAFs in the present patient population were compared with published frequencies from the Allele Frequency Aggregator (ALFA) project of the National Center for Biotechnology Information (NCBI) Database of Genotypes and Phenotypes (dbGaP) (**Table 2**) (29). Each SNP was then tested for association with the clinical endpoints of pneumonia, sepsis, and septic shock using either the chi-squared test or, if n ≤ 5, Fisher’s exact test. Two models were performed for the analysis: Model 1 analyzed differences in the absolute numbers of genetic variants [wildtype (WT), heterozygous mutation (HET), and homozygous mutation (HOM)], whereas Model 2 selected differences based on allele frequencies. The OR was calculated to determine the strength of the associations identified as statistically significant. A *post hoc* analysis with Bonferroni correction was required before calculating the OR in Model 1 for cases with homozygous SNPs.

**Table 2:** Comparison of MAFs of SNPs in our cohort with published MAFs from the ALFA project (MAF dbSNP accessed 4/1/2024 https://www.ncbi.nlm.nih.gov/snp/ALFAAlleleFrequency).

	GLOBAL	EUROPE	COHORT n=105
**rs2393799 (C-762T)**	C=0.75697 T=0.24303	C=0.82278 T=0.17722	C=0.75238 T=0.24761
**rs35933842 (G151 + 1T)**	G=0.99243 T=0.00757	G=0.99177 T=0.00823	G=0.99523 T=0.00476
**rs17525809 (T253C)**	T=0.93101 C=0.06899	T=0.92747 C=0.07253	T=0.91428 C=0.08571
**rs28360447 (G474A)**	G=0.98412 A=0.01588	G=0.98279 A=0.01721	G=0.98571 A=0.01428
**rs208294 (C489T)**	C=0.54850 T=0.45150	C=0.55220 T=0.44780	C=0.52884 T=0.47115
**rs208307 (C641-5G)**	C=0.70101 G=0.29899	C=0.69956 G=0.30044	C=0.68571 G=0.31428
**rs7958311 (G835A)**	G=0.74385 A=0.25615	G=0.74711 A=0.25289	G=0.75714 A=0.24285
**rs7958316 (G853A)**	G=0.98106 A=0.01894	G=0.97922 A=0.02078	G=0.98571 A=0.01428
**rs28360457 (G946A)**	G=0.98759 A=0.01241	G=0.98652 A=0.01348	G=0.99047 A=0.00952
**rs1718119 (G1068A)**	G=0.61770 A=0.38230	G=0.60919 A=0.39081	G=0.65048 A=0.34951
**rs2230911 (C1096G)**	C=0.91342 G=0.08659	C=0.9236 G=0.0764	C=0.92380 G=0.07619
**rs2230912 (A1405G)**	A=0.84448 G=0.15553	A=0.83368 G=0.16632	A=0.84761 G=0.15238
**rs3751143 (A1513C)**	A=0.81091 C=0.18909	A=0.81076 C=0.18924	A=0.77142 C=0.22857
**rs3751142 (G1628T)**	G=0.90755 T=0.09245	G=0.92216 T=0.07784	G=0.92380 T=0.07619
**rs1653624 (T1729A)**	T=0.98173 A=0.01827	T=0.97967 A=0.02033	T=0.98058 A=0.01941
**rs1621388 (G1772A)**	G=0.62878 A=0.37122	G=0.61737 A=0.38263	G=0.63809 A=0.36190

In addition to the analysis of individual SNPs, a combination of five SNPs (*rs208294 (C489T), rs28360457 (G946A), rs2230912 (A1405G), rs3751143 (A1513C)*, and *rs1653624* (T1729A)) (17) was studied. The SNP genotype was coded as 1, 2 or 3 according to how many copies of the minor allele they carried (1 = WT, 2 = HET, 3 = HOM). Depending on the number of individuals with each haplotype, a chi-squared or Fisher’s exact test was used to identify associations.

## 3 Results

### 3.1 Analysis of the study population

Patients with pneumonia and sepsis had significantly prolonged hospital and ICU stays compared to all patients. On average, patients spent 46.6 days in the hospital and 20.3 days in the ICU. Patients with pneumonia spent on average 55.1 days in the hospital (p=0.006) and 25.9 days in the ICU (p=0.001). Patients with sepsis spent on average 53.2 days in hospital (p=0.005) and 24.3 days in ICU (p<0.001). In contrast, septic shock patients had shorter hospital and ICU stays than the overall study population (38.5 vs. 18.7 days, respectively). Septic shock patients were older than other patients (mean age 65.92 vs. 61.06 years, respectively) and were more likely to die [45.8% vs. 27.6%, respectively, p=0.023; OR: 2.96 (1.14-7.73)]. In the entire cohort, 29 patients (27.6%) died, of which 18 patients were male (62.1%) and 11 patients were female (37.9%).

### 3.2 Calculation of the Hardy-Weinberg equilibrium and linkage disequilibrium

All 16 SNPs were first tested for deviation from HWE, with only two SNPs [*rs2230911 (C1096G)* and *rs3751142 (G1628T*)] deviating from HWE (**Table 3**). The SNPs *rs35933842 (G151 + 1T)* and *rs28360457 (G946A)* were excluded from further analysis because of a MAF <1% (**Table 3**). Most of the calculated allele frequencies were consistent with the globally published frequencies from the ALFA project (29). Using the European cohort as a reference, only minor deviations >5% were found at *rs2393799 (C- 762T)*. LD analysis showed a block with high LD between *rs7958311 (G835A), rs1718119 (G1068A), rs2230911 (C1096G), rs2230912 (A1405G), rs3751143 (A1513C), rs3751142 (G1628T)* and *rs1621388 (G1772A).* In addition, further pairwise LD existed in the investigated cohort (**Figure 3D**).

**Table 3:** Hardy-Weinberg-equilibrium (HWE) and Minor Allele Frequencies (MAFs) of the investigated SNPs.

dbSNP ID	HWE		MAF
	Chi^2^-test	p-value	
**rs2393799 (C-762T)**	1.801	0.180	0.248
**rs35933842 (G151 + 1T)**	0.002	0.961	0.005*
**rs17525809 (T253C)**	0.923	0.337	0.086
**rs28360447 (G474A)**	0.022	0.882	0.014
**rs208294 (C489T)**	0.183	0.669	0.471
**rs208307 (C641-5G)**	0.544	0.461	0.314
**rs7958311 (G835A)**	0.010	0.918	0.243
**rs7958316 (G853A)**	0.022	0.882	0.014
**rs28360457 (G946A)**	0.010	0.922	0.010*
**rs1718119 (G1068A)**	0.377	0.539	0.350
**rs2230911 (C1096G)**	10.985*	0.001*	0.076
**rs2230912 (A1405G)**	0.180	0.671	0.152
**rs3751143 (A1513C)**	1.936	0.164	0.229
**rs3751142 (G1628T)**	10.985*	0.001*	0.076
**rs1653624 (T1729A)**	0.040	0.841	0.019
**rs1621388 (G1772A)**	0.902	0.342	0.362

SNPs deviating from HWE or with an MAF <1% are highlighted in bold letter with *.

### 3.3 Association of SNPs with clinical endpoints

Four SNPs showed statistically significant associations with the clinical outcomes of pneumonia and/or sepsis, but no association with septic shock or death (**Table 4**). Two of them were characterized as LOF [*rs17525809 (T253C)*, and *rs2230911 (C1096G)*], and *rs3751142 (G1628T)* and *rs1621388 (G1772A)* are synonymous SNPs with as yet unknown effect on P2X7R function. These results suggest that genetics related to impaired P2X7R function and signaling may play a role in pneumonia and sepsis.

Table 4Presentation of the significant associations between SNPs and clinical endpoints.

**Table:** 

(A) Demonstration of associations between genetic variants with pneumonia											
SNP	Total	Genotype frequency			Model 1			Model 2			
		WT	HET	HOM	p-value	OR	95% CI	MAF	p-value	OR	95% CI
rs17525809 (T253C)											
Pneumonia	43	40	3	-	0.021	0.24	(0.06 - 0.87)	0.035	0.028	0.26	(0.07 - 0.94)
Non-pneumonia	62	47	15	-				0.121			
rs2230911 (C1096G)											
Pneumonia	43	33	8	2	0.012	7.15	(1.43 - 35.67)	0.140	0.004	4.85	(1.51 - 15.63)
Non-pneumonia	62	59	2	1				0.032			
rs3751142 (G1628T)											
Pneumonia	43	33	8	2	0.012	7.15	(1.43 - 35.67)	0.140	0.004	4.85	(1.51 - 15.63)
Non-pneumonia	62	59	2	1				0.032			
rs1621388 (G1772A)											
Pneumonia	43	21	18	4	0.318			0.302	0.135	0.64	(0.36 - 1.15)
Non-pneumonia	62	24	26	12				0.403

**Table:** 

(B) Demonstration of associations between genetic variants with sepsis											
SNP	Total	Genotype frequency			Model 1			Model 2			
		WT	HET	HOM	p-value	OR	95% CI	MAF	p-value	OR	95% CI
rs17525809 (T253C)											
Sepsis	75	68	7		0.001	0.18	(0.06 - 0.52)	0.047	0.001	0.22	(0.08 - 0.59)
Non-sepsis	30	19	11					0.183			
rs2230911 (C1096G)											
Sepsis	75	62	10	3	0.045			0.107	0.007		
Non-sepsis	30	30						0.000			
rs3751142 (G1628T)											
Sepsis	75	62	10	3	0.045			0.107	0.007		
Non-sepsis	30	30						0.000			
rs1621388 (G1772A)											
Sepsis	75	36	31	8	0.078			0.313	0.021	0.49	(0.26 - 0.90)
Non-sepsis	30	9	13	8				0.483			

The minor variant of *rs17525809 (T253C)* was linked to reduced risk of developing pneumonia [Model 1: p=0.021, OR HET/WT: 0.24 (0.06 – 0.87), Model 2: MAF cohort with pneumonia 0.035 vs. MAF cohort without pneumonia 0.121, p=0.028, OR minor/major 0.26 (0.07 – 0.94)] and sepsis [Model 1: p=0.001, OR HET/WT: 0.18 (0.06 – 0.52), Model 2: MAF cohort with sepsis 0.047 vs. MAF cohort without sepsis 0.183, p=0.001, OR minor/major: 0.22 (0.08 – 0.59)]. However, as shown in **Supplementary Figure 1**, a closer look at the data reveals that the heterozygous genotype of the LOF SNP *rs17525809 (T253C)* is more frequent in sepsis patients with pneumonia than in sepsis patients without pneumonia (6/29 vs 3/22, respectively). An imbalance in *rs17525809* (T253C) genotype frequency was also observed when comparing septic and non septic patients.

The minor variant of the LOF SNP *rs2230911 (C1096G)* was associated with a higher risk of developing pneumonia [Model 1: p=0.012, OR HET/WT: 7.15 (1.43 - 35.67), Model 2: MAF cohort with pneumonia 0.140 vs. MAF cohort without pneumonia 0.032, p=0.004, OR minor/major 4.85 (1.51 - 15.63)] and sepsis [Model 1: p=0.045, OR HET/WT: -, Model 2: MAF cohort with sepsis 0.107 vs. MAF cohort without sepsis 0.000, p=0.007, OR minor/major: -]. Furthermore, the cohort of patients with sepsis or septic shock plus pneumonia had the highest proportion of heterozygous and homozygous mutant genotypes of this SNP (see **Supplementary Figure 2**).

Subsequent LD calculation with D’=1 showed that the LOF SNP *rs2230911 (C1096G)* and the synonymous SNP *rs3751142 (G1628T)* were in strong linkage **Figure 3D**. In the current cohort of patients analyzed, every patient with a mutation in the LOF SNP *rs2230911 (C1096G)* was also mutated for *rs3751142 (G1628T)*. As our study is still ongoing, n=250 patients have now been sequenced, of which only two patients were found to differ in the aforementioned combination of SNPs (data not shown).

The present study also found an association between the major variant G of *rs1621388 (G1772A)* and a higher prevalence of sepsis cases in Model 2 [Model 1: p=0.078, Model 2: MAF cohort with sepsis 0.313 vs. MAF cohort without sepsis 0.483, p=0.021, OR minor/major 0.49 (0.26-0.90)]. The association with *rs1621388 (G1772A)* was not statistically significant after Bonferroni correction in Model 1. As shown in **Supplementary Figure 3**, the distribution of wild-type, heterozygous and homozygous mutant variants in our group of non-septic patients is almost equal between wild-type, heterozygous and homozygous mutant genotypes. Sepsis and septic shock patients had lower frequencies of the minor variant. There is no systematic change in these SNP variations when including cases with and without pneumonia. Interestingly, this SNP was in complete LD with the GOF SNP *rs1718119 (G1068A)* (**Figure 3A**).

### 3.4 SNP combinations

In addition to analyzing individual SNPs, this study also investigated associations with previously identified genotype combinations consisting of five *P2RX7* SNPs (**Figure 3B**). Analysis revealed trends between three combinations and clinical outcomes that were just above the threshold for statistical significance. #11111 and #21111 showed adverse effects for ICU patients: #11111 (n=18) was associated with higher mortality [p=0.085, OR 2.60 (0.90 - 7.48)] and #21111 (n=14) with more frequent development of pneumonia [p=0.054, OR 3.06 (0.94 - 9.89)]. In contrast, #21211 (heterozygous at rs208294 His-155>Tyr, rs22309122 Gln-460>Arg, and WT at three other SNPs) (n=14) increased the likelihood of patient survival [p=0.105, OR 0.18 (0.02 - 1.42)]. For clarity, the distribution of #11111, #21121, (heterozygous at rs208294 His-155>Tyr, and rs3751146 Glu-496>Ala, and WT at three other SNPs), #21211 and others among patient groups is shown in **Figure 3C**. 5/10 patients with #*11111* without pneumonia survived, and 5/8 with pneumonia survived. In patients with #*21121* and low ion channel function (17), 4/6 patients had pneumonia and survived and 7/12 patients had no pneumonia and survived. In patients with #*21211* and high ion channel activity (17), 4/14 patients had pneumonia of which 1 died (**Figure 3D**). In the remaining haplotypes, 24/30 patients survived without pneumonia, n=25 had pneumonia of which n=7 patients died.

## 4 Discussion

### 4.1 Clinical aspects of the sample

Several aspects of the collected clinical data and sample distribution suggest that this cohort is a representative study population. The analysis confirmed longer hospitalization and ICU stays for pneumonia and sepsis, but not for septic shock (5). Shorter ICU stays for septic shock patients are similar to published data sets with higher mortality among septic shock patients. Similar to large multicenter studies, the gender distribution in the ICU is strongly skewed towards males (73%) (30). Many patients studied here, were obese and suffered from diabetes (**Supplementary Table 1**). We found that type II diabetes ICU patients present with significantly higher numbers of inflammatory monocytes (31). Since P2X7R expression is a hallmark of inflammation, future studies should address sepsis in obesity and type II diabetes as a separate study population (31).

### 4.2 LD block

This present study revealed an LD block between seven SNPs comprising a base length of one kb. The full length of *P2RX7* is 53 kb (32). In addition, pairwise LD was observed for several SNPs. A previous study in an Australian population identified an LD block in exons 11 to 13. The Australian haplotype overlapped with the haplotype of this study for five SNPs: *rs1718119 (G1068A), rs2230911 (C1096G), rs2230912 (A1405G)*, and *rs3751143 (A1513C)*. The LD block for the sample of this study also included *rs7958311 (G835A), rs3751142 (G1628T)*, and *rs1621388 (G1772A).* The variants *rs3751142 (G1628T)* and *rs1621388 (G1772A)* were not examined in the comparative study, while the LD block did not include *rs7958311 (G835A)* despite high LD levels (8, 11).

Remarkably, mutations in the GOF SNP *rs1718119 (G1068A)* and the synonymous SNP *rs1621388 (G1772A)* were identical in all patients presenting with a heterozygous or homozygous mutated genotype but differed in other SNPs. The same was observed for the LOF SNP, *rs2230911 (C1096G)* and synonymous SNP, *rs3751142 (G1628T)*, but no homozygous mutant genotypes were identified in these two SNPs.

This strong LD between the aforementioned GOF and LOF SNPs on the one hand as well as the combined presence of minor variants of GOF and LOF SNPs in a defined individual is likely linked to the biological function of this receptor since other SNPs in this LD block are not identically mutated in a number of other patients.

### 4.3 rs17525809 (T253C)

The SNP *rs17525809 (T253C)* is located in exon 2, which encodes a part of the extracellular loop of P2X7R. The minor variant causes an exchange of valine to alanine at position 76 of the protein (11). The amino acid change of this SNP affects both ion flux and fluorescent dye uptake of the P2X7R pore. Studies in multiple sclerosis (MS) have shown that the T allele, which is more common in MS patients, results in a 1.71-fold higher calcium influx into the cytosol of transfected cells compared to P2X7R with the C allele. Thus, the SNP corresponds to a LOF variant (11, 19). Previous studies have only performed functional assays for homozygous mutations of *rs17525809 (T253C)*. Therefore, the effect of heterozygous mutations on the functionality of the receptor remains unclear.

In the current cohort, the minor variant of this SNP was identified as a heterozygous and not as a homozygous mutation. Similarly, previous publications have reported that a homozygous *rs17525809 (T253C*) variant is rare (33, 34). In our study, the minor allele of *rs17525809 (T253C*) was associated with a lower OR for pneumonia and sepsis in ICU patients. The minor allele C was linked to a 4.17-fold lower risk of developing pneumonia in model 1 and a 5.56-fold lower risk of developing sepsis. The heterozygous genotype of the LOF SNP *rs17525809 (T253C)* was also more frequent in sepsis patients with pneumonia. Furthermore, not only the pneumonia/sepsis cohorts, but also the non-sepsis group showed an imbalance of *rs17525809 (T253C)* with a higher frequency of the minor allele than in reported healthy controls. A possible explanation for this imbalance may be related to the high relative numbers of individuals with obesity. In the non-septic patients, 10/30 patients had a BMI of >30 kg/m², compared to 13/24 septic shock patients with a BMI >30 kg/m² (**Supplementary Table 1**). There is evidence for the function of P2X7R signaling in energy metabolism, fat mass and weight gain (35), and it is likely that this impacts the association between *P2RX7* SNPs and sepsis.

In previously published studies, the effect of *rs17525809 (T253C)* varies depending on the investigated pathology. In chronic inflammatory diseases such as gout or MS, the LOF variant was protective (33, 36). In the context of cervical cancer, a functional P2X7R may be beneficial for targeting tumor cells (34). Synthesizing previously study results with the findings of this study, *rs17525809 (T253C)* may be protective in severe infections such as pneumonia and early sepsis inflammasome associated illness by attenuating cytokine levels such as IL-1β and IL-18.

### 4.4 rs2230911 (C1096G)

The second SNP with a significant association was *rs2230911 (C1096G)*, which causes an amino acid exchange from threonine to serine at position 357 (11). Considering the structure of P2X7R, this amino acid exchange is located in the cytoplasmic juxtamembrane domain. This part of the receptor mediates most of the P2X7R-induced effects, including macropore formation (26, 37). Within the juxtamembrane region, *rs2230911 (C1096G)* lies in a cholesterol recognition amino acid consensus (CRAC) motif (26). Such a region has the potential for interaction with cholesterol (38). For P2X7R, cholesterol decreases ion channel and macropore opening and limits excessive receptor activation (39). Subsequently, *rs2230911 (C1096G)* leads to a reduced functionality of P2X7R in both ion channel and macropore formation (21). Several LOF SNPs individually affect the properties and signaling events of P2X7R depending on their location in the gene. For example, rs2230911 (C1096G) impacts the pore formation of the receptor, which is probably due to its location in the C-terminus (21).

In addition to the above-described location in the CRAC motif, *rs2230911 (C1096G)* is part of a threonine-serine cluster (357 TYSS 360) which has been postulated to be a potential binding motif for β-arrestin-2. This binding would lead to the internalization of the receptor which is equivalent to its degradation and inactivation (40). In the SNP association analysis, heterozygous mutation carriers for *rs2230911 (C1096G)* had a higher occurrence of pneumonia and sepsis.

Previously published reports showed a detrimental effect of the SNP in the context of various pathologies and patients carrying rs2230911 (C1096G) may be prone to develop severe infections due to predisposing comorbidities. The minor allele of *rs2230911 (C1096G)* was linked to several comorbidities including gout, hyperglycemia with an additional risk of progression to diabetes mellitus or metabolic syndrome (41), and obesity (42). Similarly, in acute mycobacterial infection, effective killing of pathogens was only possible in the presence of a well-functioning P2X7R in the absence of the *rs2230911 (C1096G)* mutation (21). These findings suggest that carrying the LOF variant *rs2230911 (C1096G)* increases the risk of developing mycobacterial infections and may be also associated with decreased clearance of lung pathogens.

### 4.5 rs3751142 (G1628T)

In this study, two synonymous SNPs showed statistically significant results for the clinical endpoints pneumonia and/or sepsis. Although the MAF of the whole cohort was consistent with published allele frequencies (**Table 2**), patients who were septic or had pneumonia had significantly higher MAF than the respective control cohorts (**Table 4**). This could be a consequence of LD with functionally relevant non-synonymous SNPs. In this cohort *rs3751142 (G1628T)* is in complete LD with the non-synonymous LOF SNP *rs2230911 (C1096G)*. Although synonymous SNPs do not lead to amino acid substitutions, changes in the nucleotide sequence of messenger ribonucleic acid (mRNA) can alter protein expression levels, protein isoform expression or protein folding (43).

The *rs3751142 (G1628T)* SNP is in exon 13 at an exonic splice enhancer site and may cause exon skipping or decrease mRNA stability leading to nonsense-mediated mRNA decay (24, 26, 44). The results presented here indicate that the sepsis cohort carried more heterozygous genotypes, and the cohort of patients suffering from sepsis plus pneumonia had the highest proportion of heterozygous and homozygously mutated genotypes at this RNA stability related SNP position. According to dbSNP *rs3751142 (G1628T)* (http://www.ncbi.nlm.nih.gov/SNP/snp_ref. cgi?locusId05027), has a MAF of 0.089, which is slightly higher in a Korean healthy donor cohort (45). Accordingly, SNP variation is low in non-septic patients, increased in patients with sepsis and septic shock, but further increased in patients with pneumonia (both sepsis and septic shock pneumonia patients).

As a relevant observation of P2X7R in sepsis, a recent study addressed the function of P2X7R in patients with early versus late sepsis, and convincingly demonstrated that P2X7R expression is upregulated in the early phase of sepsis. In contrast, in the later phases of sepsis, the P2X7R is released from the membrane and potent immunosuppressive signaling driven by the ATP degradation product adenosine is more prevalent (16). Then, non-functional P2X7R associated with *rs2230911 (C1096G)* and *rs3751142 (G1628T)* might predispose to an increased risk of pneumonia. One reason could be that P2X7R plays an important role in the lung microenvironment, such as in the paracrine regulation of surfactant exocytosis by P2X7R-positive type I alveolar epithelial cells. This is critical for the protection of alveolar barrier and fluid homeostasis (46). Thus, a functioning P2X7R may be protective in the lung, allowing ATP-inducible cytokine release, clearance of non-opsonized particles and pathogens, and promotion of immunity. Therefore, in cases at risk of pneumonia, the organ specific function of P2X7R requires higher functionality, increased and stable expression densities, that is in part provided by GOF SNPs. This would not interfere with a second signal from lung infecting pathogens and allow ATP-induced calcium influx to stimulate inflammasome activation in alveolar epithelial cells and antigen presenting cells to promote immunity. Thus, tissue localization and regulation of expression by polymorphisms in miRNA-based regulatory events (47) play an important role in coordinating inflammation and immunity.

### 4.6 rs1621388 (G1772A)

The major variant G of rs1621388 (G1772A) had a higher prevalence in sepsis cases in Model 2. The SNP r*s1621388 (G1772A)* is a synonymous SNP, that is in LD with the GOF SNP *rs1718119 (G1068A)*. The *rs1621388 (G1772A)* SNP is located at an exonic splice enhancer site but is also part of the codon for proline 582 located in an LPS binding domain in the C-terminus of P2X7R (44). Binding of LPS has been shown to lower the pore opening threshold of P2X7R (48). It is conceivable that changes in codon usage could change protein folding, altering the LPS binding site. Further investigation of the role of synonymous SNPs in P2X7R function is required to prove their functional and disease-associated properties. But it can be hypothesized that this SNP results in a GOF phenotype by lowering the threshold of P2X7R activation through the binding of endotoxin and its interaction with lipopolysaccharide binding protein (LBP) and bacterial permeability increasing protein (BPI) in the C-terminal region (44).

Future studies should address the selective contribution of gram-negative versus gram-positive bacteria for the development of sepsis in SNP *rs1621388 (G1772A)* genotypes, especially since this SNP is in LD with the GOF SNP *rs1718119 (G1068A)*.

*rs1718119 (G1068A)* has previously been shown to increase inflammasome activation through P2X7R activation (11). The trend observation of likely inferior P2X7R activation and signaling in patients with sepsis, septic shock with and without pneumonia should be noted.

### 4.7 Previously identified *P2RX7* genotypes

Combination SNP analysis in the current study did not reveal a statistically significant result. However, several trends emerged: Firstly, patients with #*11111* were 2.6 times more likely to die [p=0.085, OR 2.60 (0.90 - 7.48)]. This SNP combination consists of five wild-type SNPs indicating a highly functional P2X7R. This is linked to high sensitivity to its ligand ATP, pore formation, induction of apoptosis and regulation of phagocytosis (49, 50). Secondly, the combination #*21111* contains a heterozygous mutation of *rs208294 (C489T)* with simultaneous presence of four wild-type SNPs at *rs28360457 (G946A), rs2230912 (A1405G), rs3751143 (A1513C)*, and *rs1653624 (T1729A)*. #*21111* was associated with a 3.1-fold higher incidence of pneumonia [(p=0.054, OR 3.06 (0.94 - 9.89)]. The previously cited study in a large MS patients cohort showed that the calcium influx in variants encoded by the minor allele A of *rs208294 (C489T)*, is 3.82 higher than those encoded by the G allele of this SNP (36).

These results correspond to the highest ion flux linked to genotype #21211, which includes a heterozygous mutation of the GOF SNP *rs208294 (C489T)*, and the partial LOF SNP *rs2230912 (A1405G)* (17). These results are consistent with other studies documenting enhanced receptor function by the *rs208294 (C489T)* polymorphism even in heterozygous individuals (51, 52). This combination showed a tendency to an increased likelihood of patients’ survival [p=0.105, OR 0.18 (0.02 - 1.42)].

In addition to trauma and sepsis, chemotherapy of tumors is linked to massive ATP release and P2X7R activation. A recent validation study on functional SNPs and outcome after chemotherapy in colorectal carcinoma showed that the *P2RX7* GOF SNP *rs208294 (C489T)* was associated with worse overall survival (OS) and progression free survival, however the *P2RX7* LOF SNP *rs2230911 (C1096G)* was prognostic and predictive, showing improved OS in patients who received oxaliplatin (53). The heterozygous genotype of the LOF *rs17525809 (T253C)* -TC was shown to be associated with higher expression of the gene than wildtype TT genotype (54).

### 4.8 Conclusion

In conclusion, this study provides evidence for the functional relevance of P2X7R to combat sepsis and pneumonia, a severe combination of acquired immune deficiency and life threatening infection. A functionally reduced *P2RX7* variant has been identified by the presence of LOF SNPs. Whereas LOF SNPs may be protective against the detrimental effects of hypercytokinemia and excessive systemic inflammation, including neuroinflammation (55, 56), a SNP-defined and well-functioning P2X7R may be necessary and protective for lung infections and pneumonia by supporting ATP-inducible cytokine release, clearance of pathogens, and promotion of immunity. The LOF SNP *rs17525809 (T253C)*, and synonymous SNP, *rs1621388 (G1772A)*, appear to be linked to a lower OR for both sepsis and pneumonia for the former and sepsis alone for the latter SNP, whereas the LOF SNP *rs2230911 (C1096G)* and synonymous SNP, *rs3751142 (G1628T)*, that is related to exon skipping and splice variant expression are linked to higher OR especially in pneumonia. Further evidence for the relevance of SNP combinations is provided by the identification of a previously reported SNP genotype #21211 [17] which is more frequent in sepsis survivors. To better understand the genetic implications of the P2X7R and identify conditions which improve by blocking or activating P2X7R, functional assays with patient-derived macrophages may be helpful (57). in addition, the time course of sepsis as a clinical condition is of relevance to appropriately address P2X7R as a therapeutic target (16). Further studies might concentrate on longitudinal observational studies including the documentation of comorbidities as well as early and late states of sepsis, septic shock, the manifestation of acute respiratory distress syndrome, and pneumonia. These clinical states should be characterized by inflammatory biomarkers as well as levels of plasma ATP, soluble P2X7R and sCD14 including functional immune capacity tests.

### 4.9 Limitations of the study

The study results are to be interpreted in the context of several limitations, which were mostly of a structural type. Above all, the sample size of n = 105 patients limited the creation of subgroups described by more detailed clinical information. Additionally, the clinical data did not sufficiently include the pre-existing diseases and conditions of the patients despite the critical roles such pre-morbidities play in clinical outcomes (58). In the context of likely different *P2RX7* genotypes linked to functional phenotypes that manifest in sepsis versus successful control of respiratory diseases, a future clinical study should be initiated to selectively compare cases with pneumonia independently from sepsis. A focus of this study should be to document *P2RX7* genotypes, inflammasome activation, and pathogen control. Moreover, future studies need to account for LD between different SNPs and thus differentiate between association and causality of findings.

Despite the above-described limitations, this study provides some evidence on the extent to which genetic variants have a protective or detrimental effect in critically ill patients.

## Author’s note

According to recent findings: Zorina-Lichtenwalter, K., A. R. Ase, V. Verma, A. I. M. Parra, S. Komarova, A. Khadra, P. Séguéla and L. Diatchenko (2024). "Characterization of Common Genetic Variants in P2RX7 and Their Contribution to Chronic Pain Conditions." The Journal of Pain 25(2): 545-556. P2RX7 GOF and LOF functions and genotypes should be distinguished by their effects on channel opening and pore formation which may require functional assays of SNP-genotyped patients‘ derived cells and tissues.

## Data availability statement

The original contributions presented in the study are publicly available. This data can be found here: https://oparu.uni-ulm.de/items/12deb668-2371-41f6-b77a-115d8af31887.

## Ethics statement

The studies involving humans were approved by Ulm University, Ethics committee. The studies were conducted in accordance with the local legislation and institutional requirements. Written informed consent for participation in this study was provided by the participants’ legal guardians/next of kin.

## Author contributions

JG: Investigation, Writing – original draft. SF: Conceptualization, Funding acquisition, Investigation, Writing – original draft. KS: Methodology, Investigation, Writing – review & editing. BM: Methodology, Writing – review & editing, Project administration. ES: Conceptualization, Methodology, Project administration, Supervision, Writing – original draft.
