# Supplementary material for: Loss-of-function/gain-of-function polymorphisms of the ATP sensitive P2X7R influence sepsis, septic shock, pneumonia, and survival outcomes
Source: Front Immunol. 2024 Jun 20;15:1352789. doi: 10.3389/fimmu.2024.1352789 (PMC11222724; doi:10.3389/fimmu.2024.1352789)
Supplement: Supplementary file 2 [file DataSheet_1.docx]

Novel loss-of-function (LOF) polymorphisms of the ATP sensitive P2X7 dissect patients with sepsis, septic shock, pneumonia

**Johanna Guggemos^1^, Stephen J. Fuller^2,3^, Kristy K. Skarratt^2,3^, Benjamin Mayer^4^ and E. Marion Schneider^1*^**

^1^Anesthesiology and Intensive Care Medicine, Ulm University Hospital, Ulm, Germany

^2^ Nepean Clinical School, Faculty of Medicine and Health, The University of Sydney, Kingswood, NSW, Australia

^3^Nepean Hospital, Penrith, NSW, 2750 Australia

^4^Institute for Epidemiology and Medical Biometry, Ulm University, Ulm, Germany

*** Correspondence:**E. Marion Schneider

Clinic of Anaesthesiology and Intensive Care Medicine

Ulm University Hospital

Albert-Einstein-Allee 23

89081 Ulm

[https://orcid.org/0000-0003-2750-3399](https://orcid.org/0000-0003-2750-3399?lang=en)

Marion.Schneider@uniklinik-ulm.de

Supplementary files


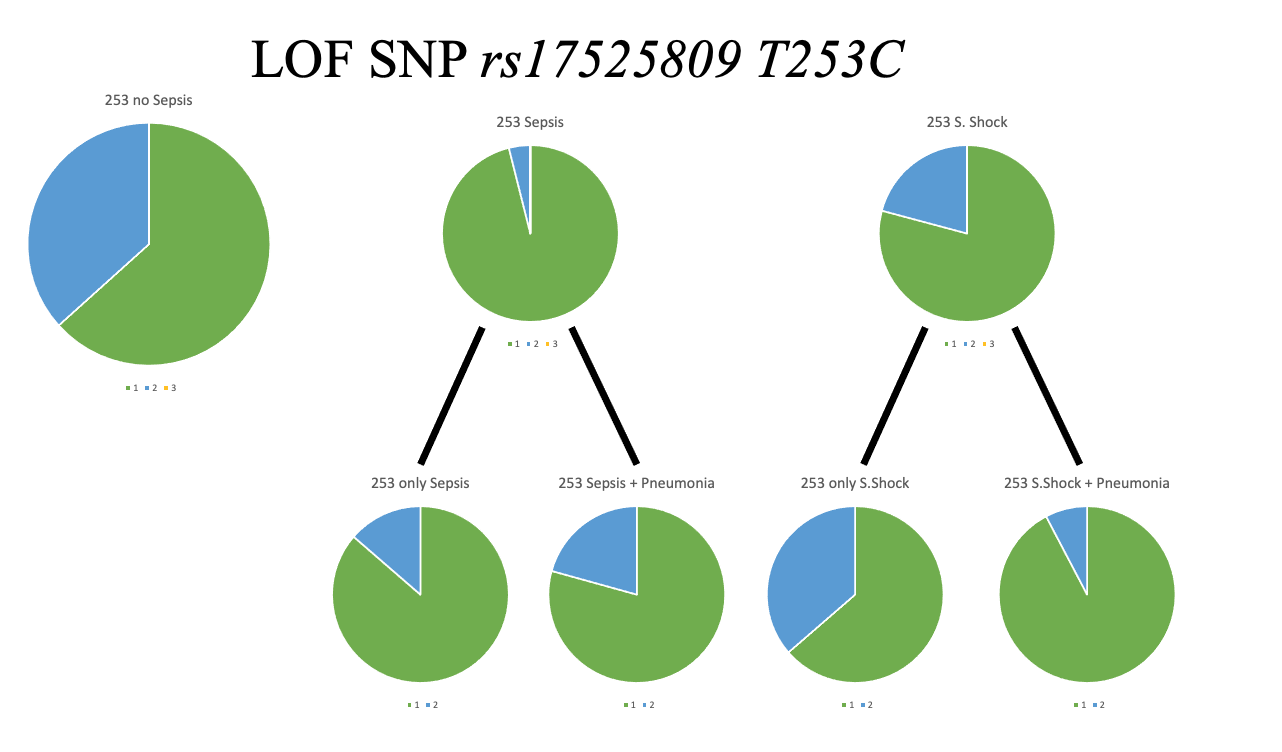


**Supplementary Figure 1.** Genotype distribution of LOF SNP *rs17525809 (T253C)* in non-sepsis, sepsis and septic shock patients with and without pneumonia. Wildtype genotypes are colored green and heterozygous genotypes blue.


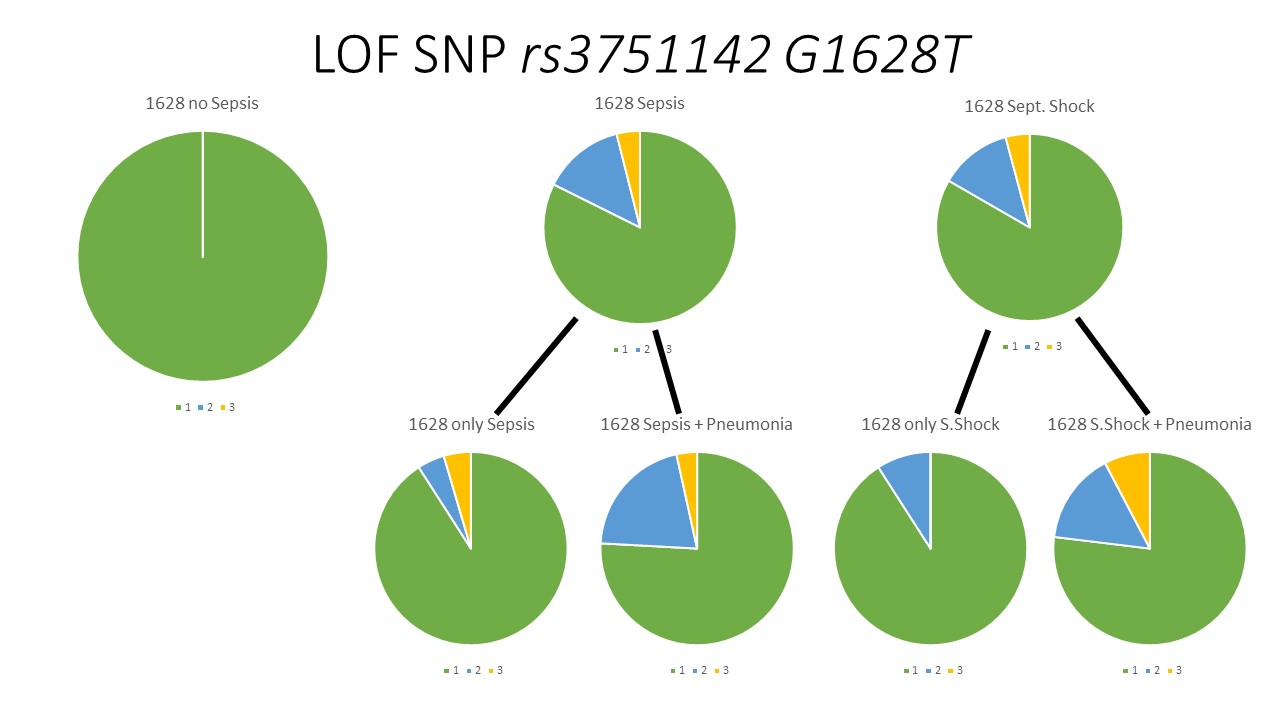


**Supplementary Figure 2.** Distribution of genotypes for P2RX7 RNA stability SNP *rs3751142* (*G1628T*) which is in complete linkage with the LOF SNP *rs2230911 (C1096*G), in non-septic patients, patients with sepsis and septic shock (upper row) as well as patients with the additional diagnosis of pneumonia (lower row). Wildtype genotypes are shown as green parts of the cake diagram, heterozygous genotypes (blue) and homozygously mutated genotypes (yellow).


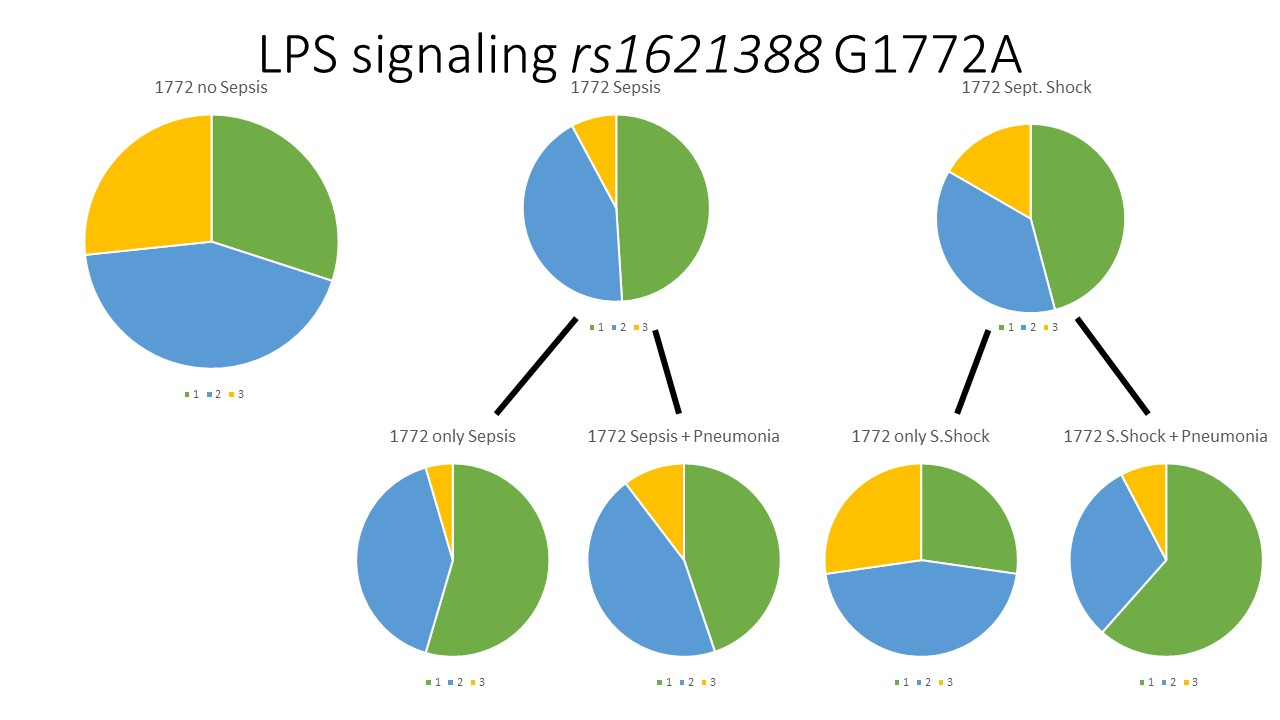


**Supplementary Figure 3.** Distribution of genotypes for P2XR7 SNP rs1621388 G1772A in non-septic patients, patients with sepsis and septic shock (upper row) as well as patients with the additional diagnosis of pneumonia (lower row). Wildtype genotypes are shown as green parts of the cake diagram, heterozygous genotypes (blue) and homozygously mutated genotypes (yellow).

Distribution of Risk Haplotypes of P2X7 in patients without sepsis, with sepsis and septic shock

|  |  | |  | | |  |  |  |  |  |  |  |  |
| --- | --- | --- | --- | --- | --- | --- | --- | --- | --- | --- | --- | --- | --- |
| 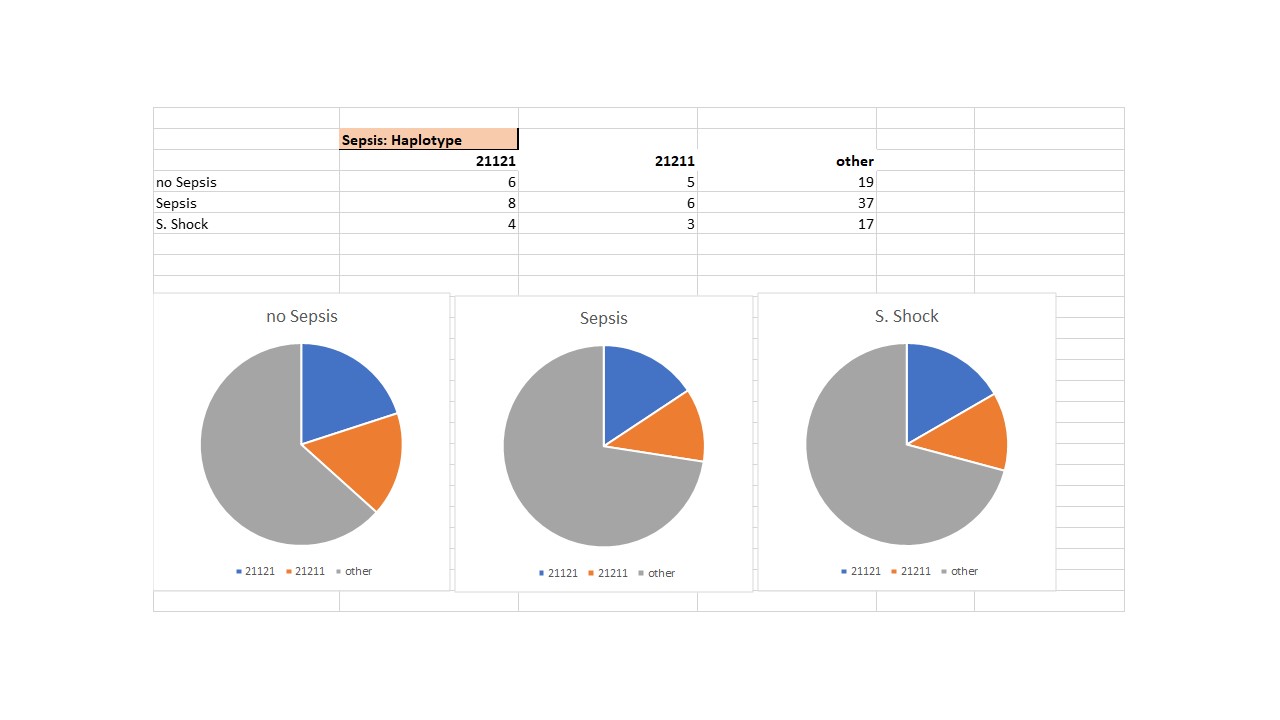 | | | | | | | | |  |  |  |  |  |
|  | | | | | | | | |  |  |  |  |  |
|  | |  | |  |  |  |  |  |  |  |  |  |  |

**Supplementary Figure 4.** Haplotype distribution of #*21121* with low P2X7 ion channel function, and #*21211* with high P2X7 ion channel function amongst patients without sepsis, sepsis and septic shock is similar.

|  |  | **Pneumonia** | | | | **Sepsis** | | | | **Septic shock** | | | | **Death** | | | |
| --- | --- | --- | --- | --- | --- | --- | --- | --- | --- | --- | --- | --- | --- | --- | --- | --- | --- |
|  |  | **MAF Pneumonia** | |  |  | **MAF Sepsis** | |  |  | **MAF septic Shock** | |  |  | **MAF Death** | |  |  |
|  | **MAF** | yes | no | p-value | OR (CI) | yes | no | p-value | OR (CI) | yes | no | p-value | OR (CI) | yes | no | p-value | OR (CI) |
| **rs2393799 (C-762T)** | 0.248 | 0.233 | 0.258 | 0.674 | 0.87  (0.46 - 1.66) | 0.233 | 0.283 | 0.448 | 0.77  (0.39 - 1.52) | 0.271 | 0.241 | 0.671 | 1.17  (0.56 - 2.43) | 0.310 | 0.224 | 0.193 | 1.56  (0.80 - 3.07) |
| **rs17525809 (T253C)** | 0.086 | 0.035 | 0.121 | **0.028** | 0.26  (0.07 - 0.94) | 0.047 | 0.183 | **0.001** | 0.22  (0.08 - 0.59) | 0.104 | 0.080 | 0.603 | 1.33  (0.45 - 3.95) | 0.138 | 0.066 | 0.095 | 2.27  (0.85 - 6.06) |
| **rs28360447 (G474A)** | 0.014 | 0.012 | 0.016 | 1.000 | 0.72  (0.06 - 8.06) | 0.007 | 0.033 | 0.203 | 0.20  (0.02 - 2.24) | 0.000 | 0.019 | 1.000 | - | 0.000 | 0.020 | 0.563 | - |
| **rs208294 (C489T)** | 0.471 | 0.465 | 0.475 | 0.884 | 0.96  (0.55 - 1.67) | 0.473 | 0.466 | 0.919 | 1.03  (0.56 - 1.89) | 0.479 | 0.469 | 0.899 | 1.04  (0.55 - 1.99) | 0.431 | 0.487 | 0.471 | 0.80  (0.43 - 1.47) |
| **rs208307 (C641-5G)** | 0.314 | 0.244 | 0.363 | 0.068 | 0.57  (0.31 - 1.05) | 0.280 | 0.400 | 0.091 | 0.58  (0.31 - 1.09) | 0.396 | 0.290 | 0.166 | 1.60  (0.82 - 3.13) | 0.345 | 0.303 | 0.556 | 1.21  (0.64 - 2.30) |
| **rs7958311 (G835A)** | 0.243 | 0.233 | 0.250 | 0.772 | 0.91  (0.48 - 1.73) | 0.267 | 0.183 | 0.203 | 1.62  (0.77 - 3.42) | 0.229 | 0.247 | 0.801 | 0.91  (0.42 - 1.94) | 0.241 | 0.243 | 0.975 | 0.99  (0.49 - 2.00) |
| **rs7958316 (G853A)** | 0.014 | 0.023 | 0.008 | 0.122 | 7.41  (0.66 - 83.33) | 0.013 | 0.017 | 1.000 | 0.80  (0.07 - 8.93) | 0.000 | 0.019 | 1.000 | - | 0.017 | 0.013 | 1.000 | 1.32  (0.12 - 14.71) |
| **rs1718119 (G1068A)** | 0.350 | 0.302 | 0.383 | 0.229 | 0.70  (0.39 - 1.26) | 0.313 | 0.446 | **0.075** | 0.57  (0.30 - 1.06) | 0.354 | 0.348 | 0.938 | 1.03  (0.52 - 2.02) | 0.345 | 0.351 | 0.930 | 0.97  (0.51 - 1.84) |
| **rs2230911 (C1096G)** | 0.076 | 0.140 | 0.032 | **0.004** | 4.85  (1.51 - 15.63) | 0.107 | 0.000 | **0.007** | - | 0.104 | 0.068 | 0.371 | 1.60  (0.53 - 4.85) | 0.052 | 0.086 | 0.565 | 0.58  (0.16 - 2.13) |
| **rs2230912 (A1405G)** | 0.152 | 0.140 | 0.161 | 0.666 | 0.84  (0.39 - 1.83) | 0.147 | 0.167 | 0.716 | 0.86  (0.38 - 1.94) | 0.146 | 0.154 | 0.886 | 0.94  (0.38 - 2.32) | 0.086 | 0.178 | 0.099 | 0.44  (0.16 - 1.19) |
| **rs3751143 (A1513C)** | 0.229 | 0.198 | 0.250 | 0.375 | 0.74  (0.38 - 1.44) | 0.220 | 0.250 | 0.640 | 0.85  (0.42 - 1.70) | 0.146 | 0.253 | 0.120 | 0.50  (0.21 - 1.21) | 0.190 | 0.243 | 0.407 | 0.73  (0.34 - 1.55) |
| **rs3751142 (G1628T)** | 0.076 | 0.140 | 0.032 | **0.004** | 4.85  (1.51 - 15.63) | 0.107 | 0.000 | **0.007** | - | 0.104 | 0.068 | 0.371 | 1.60  (0.53 - 4.85) | 0.052 | 0.086 | 0.565 | 0.58  (0.16 - 2.13) |
| **rs1653624 (T1729A)** | 0.019 | 0.024 | 0.016 | 1.000 | 1.46  (0.20 - 10.64) | 0.020 | 0.017 | 1.000 | 1.18  (0.12 - 11.63) | 0.042 | 0.013 | 0.232 | 3.39  (0.46 - 25.00) | 0.036 | 0.013 | 0.300 | 2.74  (0.38 - 20.00) |
| **rs1621388 (G1772A)** | 0.362 | 0.302 | 0.403 | 0.135 | 0.64  (0.36 - 1.15) | 0.313 | 0.483 | **0.021** | 0.49  (0.26 - 0.90) | 0.354 | 0.364 | 0.899 | 0.96  (0.49 - 1.88) | 0.345 | 0.368 | 0.750 | 0.90  (0.48 - 1.70) |

**Supplementary Table 2**: Comparison of the minor allele frequencies (MAFs) of the particular polymorphisms between patients with and without the clinical endpoints pneumonia, sepsis, septic shock, and death.
